# QTL for the Kinematic Traits That Define the Arabidopsis Root Elongation Zone and Their Relationship to Gravitropism

**DOI:** 10.3390/plants13091189

**Published:** 2024-04-25

**Authors:** Ashley R. Henry, Nathan D. Miller, Edgar P. Spalding

**Affiliations:** Department of Botany, University of Wisconsin, Madison, WI 53706, USA; arhenry2@wisc.edu (A.R.H.); ndmiller@wisc.edu (N.D.M.)

**Keywords:** cell expansion, root growth, elongation zone, kinematics, QTL

## Abstract

Cell expansion in a discrete region called the elongation zone drives root elongation. Analyzing time lapse images can quantify the expansion in kinematic terms as if it were fluid flow. We used horizontal microscopes to collect images from which custom software extracted the length of the elongation zone, the peak relative elemental growth rate (REGR) within it, the axial position of the REGR peak, and the root elongation rate. Automation enabled these kinematic traits to be measured in 1575 Arabidopsis seedlings representing 162 recombinant inbred lines (RILs) derived from a cross of Cvi and Ler ecotypes. We mapped ten quantitative trait loci (QTL), affecting the four kinematic traits. Three QTL affected two or more traits in these vertically oriented seedlings. We compared this genetic architecture with that previously determined for gravitropism using the same RIL population. The major QTL peaks for the kinematic traits did not overlap with the gravitropism QTL. Furthermore, no single kinematic trait correlated with quantitative descriptors of the gravitropism response curve across this population. In addition to mapping QTL for growth zone traits, this study showed that the size and shape of the elongation zone may vary widely without affecting the differential growth induced by gravity.

## 1. Introduction

Root elongation begins at germination and continues thereafter to increase the organ system that anchors the plant, absorbs water and minerals, interacts with microorganisms, and performs other functions. Root elongation depends on mitotic cell divisions in the apical meristem, but those divisions per se do not appreciably lengthen the root. Instead, new cells slowly enter the elongation zone from the root apical meristem. Upon traversing a boundary, they expand prodigiously before exiting equally abruptly into a region of differentiation and maturation. The expansion phase is severely compressed spatiotemporally, meaning that cells within a short region expand at fast rates for a short time [1,2,3,4]. Beemster and Baskin [5] used kinematic concepts and image analysis techniques to quantify the dynamics of this process in the primary root of Arabidopsis seedlings. They found that slow expansion over 4 d pushes newly born cells into an elongation phase that lasts only 6 h.

The general features of the root growth engine were known at least since Sachs [6] observed the dramatically unequal displacement of ink dots placed on the growing root of a *Vicia* seedling. The modern way to characterize root elongation in kinematic terms is to acquire a time series of digital images of a growing root and use a computer program to match the microscopic features within many small regions of an image from frame to frame. The distance each matched region moved between frames is converted into an axial velocity. From the perspective of an observer at the root tip, nearby regions move away very slowly while regions at the basal end of the elongation zone move away the fastest because the expanding material is in the interval. From a plot of velocity versus distance from the tip, the axial distribution of relative elemental growth rate (REGR) can be determined [7]. This plot defines the size, shape, and location of the elongation zone. The software Van der Weele et al. [8] created and applied to the kinematic analysis of Arabidopsis root growth showed that REGR rises beginning at a point approximately 0.2 mm basal to the quiescent center of the meristem. REGR peaks at approximately 40% h^−1^ somewhere within the most apical millimeter and then declines to near zero within the next 0.5 mm of root length. The measurements showed that cytokinin slowed the growth of the *stunted plant 1* (*stp1*) mutant by shortening the elongation zone and reducing maximum REGR [9]. Treating *stp1* with the cytokinin zeatin rescued the kinematic defects and restored the overall growth rate. A similar software application explained the kinematic basis of the cytokinin-dependent slow growth of the *auf1* mutant [10] and demonstrated that the maximum REGR of *feronia* mutants oscillates between extremes as high as 100% h^−1^ and more typical values in the range of 40% h^−1^ [11]. Stripflow [12] and GrowthTracer [13] algorithms introduced different ways to extract kinematic parameters from pairs of Arabidopsis root images. Most recently, Henry et al. [14] created the Patch Track program for kinematic analyses of Arabidopsis root growth and used it to show how three different auxin transporters affect the size, shape, and position of the Arabidopsis root elongation zone. The various technical platforms assembled to quantify the Arabidopsis root elongation zone in kinematic terms differ mostly in the method of detecting the displacement of patches of image texture, and in their degree of automation. The latter determines measurement throughput and therefore the scale of the experiment that is feasible. Henry et al. [14] increased throughput by using five horizontal microscopes each equipped with a computer-controlled camera that acquired images every 30 s for 1 h. The Patch Track software was designed to process the 120 frames and return the length of the elongation zone, the peak value of REGR within it, the axial position of this peak, and the overall elongation rate of the root. The throughput of image acquisition and the largely automated analysis made it possible to use this platform to treat the kinematic features of the Arabidopsis root elongation zone as phenotypes to measure in a population of recombinant inbred lines (RILs) for the purpose of measuring the quantitative trait loci (QTL) controlling them. This is the first major component of the present study.

The second major component of this study relates the kinematics findings to the root’s growth response to a gravity signal, i.e., gravitropism. The gravity signaling mechanism establishes a difference in cell expansion between the upper and lower sides of the elongation zone to reorient the root tip after a plant is tilted [15,16], which must involve adjustments to one or more kinematic parameters. Moore et al. [17] mapped gravitropism QTL in the same population of RILs used in this study of growth zone kinematic traits. Knowing how gravitropism response curves and elongation zone traits naturally vary in the same population enabled analyses of correlations that produced new information about the relationship between the kinematic features of a vertical elongation zone and the adjustments that produce a bend.

## 2. Results

**Figure 1:**
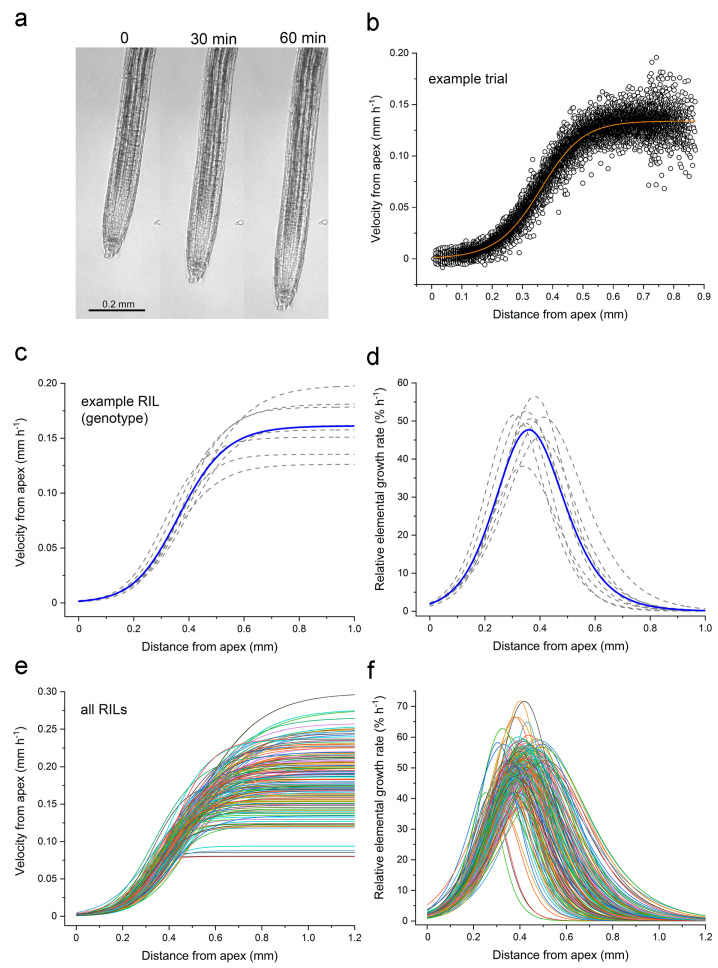
Overview of kinematic data collection starting with example images of roots and ending with a population of velocity and relative elemental growth rate profiles obtained from Cvi × L*er* recombinant inbred lines. (**a**) Three images selected from the beginning, middle, and end of a typical 120-frame trial. (**b**) Velocity point cloud showing the spatial distribution of displacement velocities of one root measured every 30 s for 1 h. The orange line shows Equation (1) (the flexible logistic function) fitted to the data. (**c**) Fitted velocity curves for 7 replicates of an arbitrarily selected RIL (dotted lines). The blue line shows the average velocity profile. (**d**) REGR profiles for the 7 trials shown in C. The blue line shows the average REGR profile. (**e**) Average velocity profile of the RILs in the Cvi × L*er* population, each plotted in a different color. (**f**) REGR profiles derived from each RIL’s average velocity profile.

The germplasm used in this quantitative genetic study of the root growth zone was a population of 162 recombinant inbred lines derived from a cross between Cvi and L*er* [18]. Horizontally mounted microscopes equipped with computer controlled digital cameras were used to collect images of individual roots growing vertically. Figure 1a shows a representative root at the beginning, midpoint, and end of the 60 min recording period. Supplemental Video S1 shows a representative root elongating for 30 min.

Each image time series was analyzed with custom software that tracked the displacement of image patches to quantify material velocity (*v*) as a function of position along the root axis (*x*), hereafter the velocity profile [14]. Figure 1b shows an example of a velocity profile for a single root measured for 1 h, and the best fit of Equation (1)

(1) vx=vf[1+e−kx−x0]1n

which is the flexible logistic function that Morris and Silk [4] developed for the kinematic analysis of velocity profiles. Figure 1c displays velocity profiles obtained in independent trials of an arbitrarily selected RIL to show typical variability. The average curve also displayed in Figure 1c was created by averaging the fitted parameters of the individual trials for that RIL. Taking the first derivative of the velocity profiles in Figure 1c produces the relative elemental growth rate (REGR) curves shown in Figure 1d. The average velocity and REGR profiles for each RIL in the Cvi × L*er* RIL population (Figure 1e,f) demonstrate that a large amount of kinematic variation exists in the elongation zone traits represented in this population.

The four parameters in Equation (1) (*v*_f_, *x*_0_, *k*, and *n*) determine the shape of REGR profiles such as those in Figure 1d,f. Supplemental Figure S1 shows how sweeping the values of these parameters changes the shape of the velocity profiles, and therefore the shape of the REGR profile, which is also the shape of the elongation zone.

**Figure 2:**
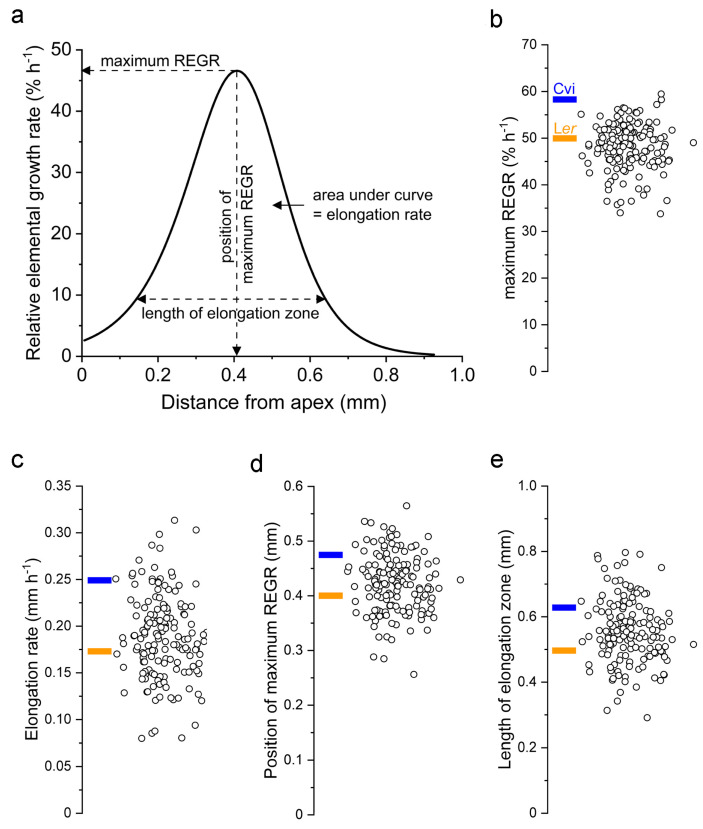
Kinematic traits of the root elongation zone and their variation across the Cvi × L*er* RIL population. (**a**) A representative relative elemental growth rate (REGR) curve labeled with the four kinematic traits of the elongation zone studied here. (**b**) The values of maximum REGR obtained for the RIL population. The blue and orange bars indicate the mean value of the inbred parents. (**c**) Same as (**b**) but for elongation rate. (**d**) Position of maximum REGR. (**e**) Length of elongation zone.

After fitting Equation (1) to the velocity profiles measured in 7–10 roots per RIL, the parameters that produced the best fit were averaged. The curve produced by these average parameters is a kinematic description of that RIL’s elongation zone. From this curve, four traits that describe the zone in terms that have biological meaning were extracted: root elongation rate, maximum REGR, axial position of the maximum REGR value, and the length of the growth zone (Figure 2a). Figure 2b shows the distributions of the traits for the population. Each point is the average value for a different RIL, with the values of the parents indicated on the graphs with blue and orange bars. Compared to L*er*, Cvi roots displayed a greater elongation rate, longer elongation zone, higher maximum REGR, and a more basal (further from the tip) maximum REGR. Transgressive segregation was observed in the population, i.e., several RILs displayed greater or lesser values for the traits than the parents. The one possible exception is the maximum REGR trait (Figure 2b). The value for the Cvi parent (57.7% h^−1^) is very close to the greatest value measured in any RIL, indicating that 60% h^−1^ may be the limit achievable by recombination between the two genomes. The length of the elongation zones and the maximum REGR varied the most in this population, ranging from 0.25 to 0.8 mm and 32 to 60% h^−1^, respectively. The elongation rate and position of the maximum REGR ranged from 0.08 to 0.32 mm h^−1^ and from 0.25 to 0.57 mm, respectively. The dataset of growth zone traits presented in Figure 2 served as phenotype data for a quantitative trait locus (QTL) mapping experiment.

### 2.1. Quantitative Trait Loci Map

**Figure 3:**
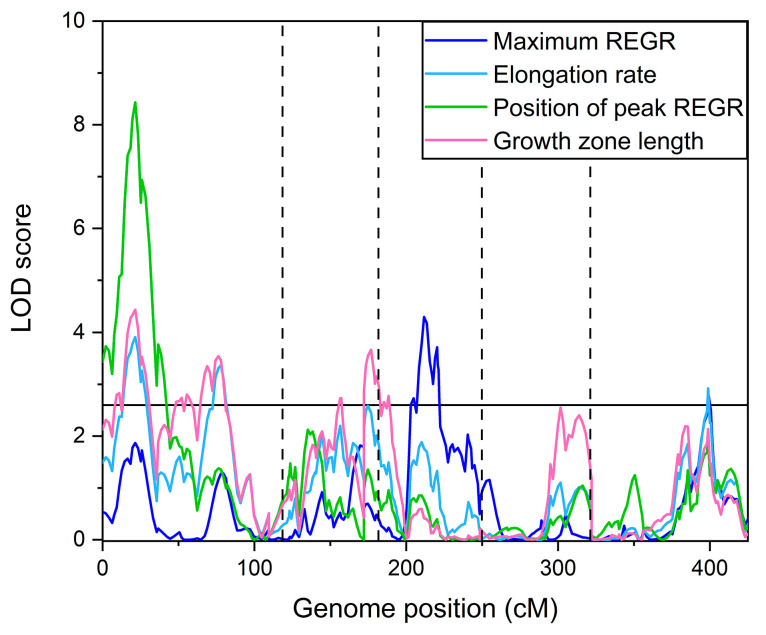
QTL map for each of the four kinematic traits of the elongation zone. The horizontal line is a significance threshold determined by the permutation method. The vertical dashed lines denote the boundaries of the five chromosomes.

Figure 3 shows the logarithm of odds (LOD) score plotted versus genome position calculated for each of the measured traits, and a significance threshold line determined by the permutation method [19] to indicate peaks that can be considered significant. Table 1 summarizes the significant QTL identified. Some loci affected more than one trait. A locus on chromosome 1 at 21.5 cm (1@21.5) was a QTL for elongation zone length, the position of maximum REGR, and elongation rate. QTL 5@76.7 affected both elongation zone length and maximum REGR, etc. The QTL 1@76.2 for the length of the elongation zone appears to coincide with a QTL for root length that Vaughn and Masson [20] detected in one of their trials using the same population.

The QTL in Table 1 explained a substantial portion of the variance in measured in the four growth zone traits. Specifically, 27% of the variance in the length of the elongation zone, 20% of the variance in the position of the maximum REGR, 19% of the variance in the elongation rate, and 16% of the variance in the maximum REGR were explained by the QTL model.

**Figure 4:**
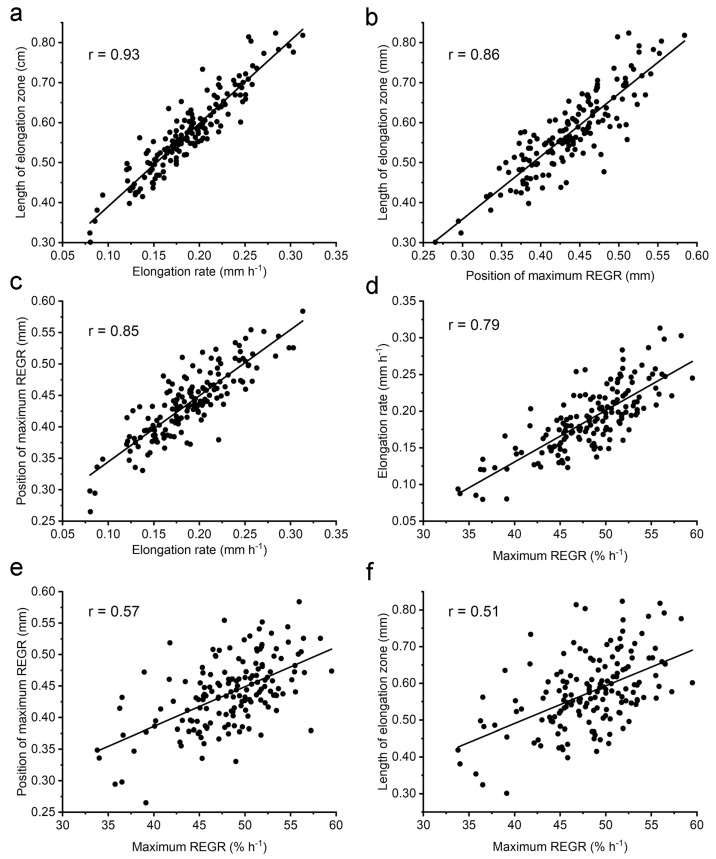
Correlation of each possible combination of four kinematic traits and the *r* value determined by a linear fit. (**a**) Length of elongation zone versus elongation rate. (**b**) Length of elongation zone versus position of maximum REGR. (**c**) Position of maximum REGR versus elongation rate. (**d**) Elongation rate versus maximum REGR. (**e**) Position of maximum REGR versus maximum REGR. (**f**) Length of elongation zone versus maximum REGR.

The fact that some QTL affected more than one kinematic trait prompted an investigation of correlation between the kinematic traits. Each pairwise comparison of the average values for each RIL is shown in Figure 4. Pearson’s correlations (*r*) between each pair of growth zone traits ranged from a high of 0.93 when the length of the elongation zone was compared to the rate of elongation (Figure 4a) to a low of 0.51 when the length of the elongation zone was compared to the maximum REGR (Figure 4f). Despite the high correlation between the rate of elongation and length of the elongation zone, intervals of the most prominent QTL for these traits did not overlap. For example, elongation rate but not the length of the elongation zone was mapped to 5@76.7. Conversely, the length of the elongation zone mapped to 2@47.6 and 3@4.8 but the elongation rate did not.

The correlation analysis made possible by the large natural variation in this population produced some observations that have straightforward explanations, such as a strong relationship between the elongation rate and length of the elongation zone (Figure 4a), while some may not have been expected. Elongation rate might be expected to correlate with the length of the elongation zone because a root with more material capable of expanding should elongate faster than one with less. It is not as clear why roots with a long elongation zone tend to have a higher capacity to expand (maximum REGR) as shown in Figure 4f. By considering those results with the plot in Figure 4e, one can infer that it is possible to have a high REGR peak in a long elongation zone, with that peak being away from the tip, but something apparently prevents short elongation zones from having a high REGR peak close to the tip.

### 2.2. Relationship of Kinematic Traits to Gravitropism

The results presented in Figure 2 and Figure 3 describe natural variation in the kinematic characteristics of the material comprising the root’s elongation zone when it is maintained vertically and not purposefully presented with any stimuli that would cause growth to change. In nature, environmental factors such as the gravity vector steer growth by creating a slower rate of elongation on one side of the root compared to the other. One or more kinematic parameters must change on one side of the root compared to the other to create differential elongation during gravitropism. The next section of this study focused on determining how the traits mapped here relate to the creation of differential elongation that changes the direction of root growth.

**Figure 5:**
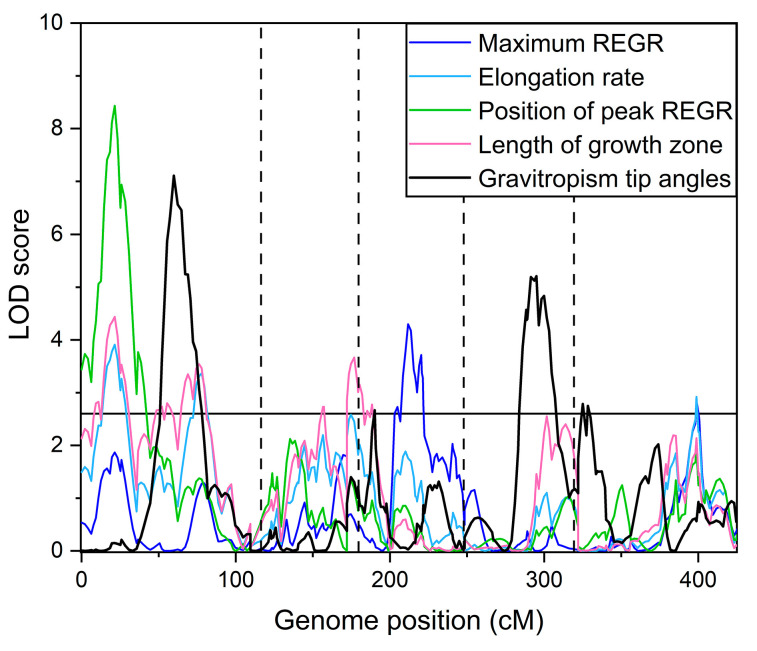
QTL map of the four elongation zone (kinematic) traits and tip angle during gravitropism from a previously published study [17]. The tip angle data represent the 4 h point of an 8 h time course. The QTL intervals for elongation zone traits overlap very little with the gravitropism QTL intervals.

Moore et al. [17] previously determined the genetic architecture of the gravitropism response by measuring the root tip angle every 2 min for 8 h following a 90° rotation in the same Cvi × L*er* RIL population used in the present study. Moore et al. [17] mapped the tip angle QTL at each of the 241 time points. The experiment was repeated with seeds from a later self-generation of the same RILs. The results of the two experiments agreed in some respects and differed in others, probably because maternal effects on seeds affect gravitropism [21,22] and environmental effects during the different recording periods could not be completely controlled. The strongest similarity between the two datasets occurred about 4 h after rotation, so we used data at this time point from the first experiment to compare the genetic architecture of the gravitropism response with the presently determined kinematic results. The goal was to determine if kinematic traits in a vertically growing root determine differential elongation during gravitropism. Figure 5 shows that the QTL maps for the kinematic traits and tip angle at the 4 h point during gravitropism had little in common. The major peaks did not overlap. Only the kinematic trait intervals centered at 1@77 and 3@4.8 overlapped to a limited extent with gravitropism QTL 1@64 and 3@17.

**Figure 6:**
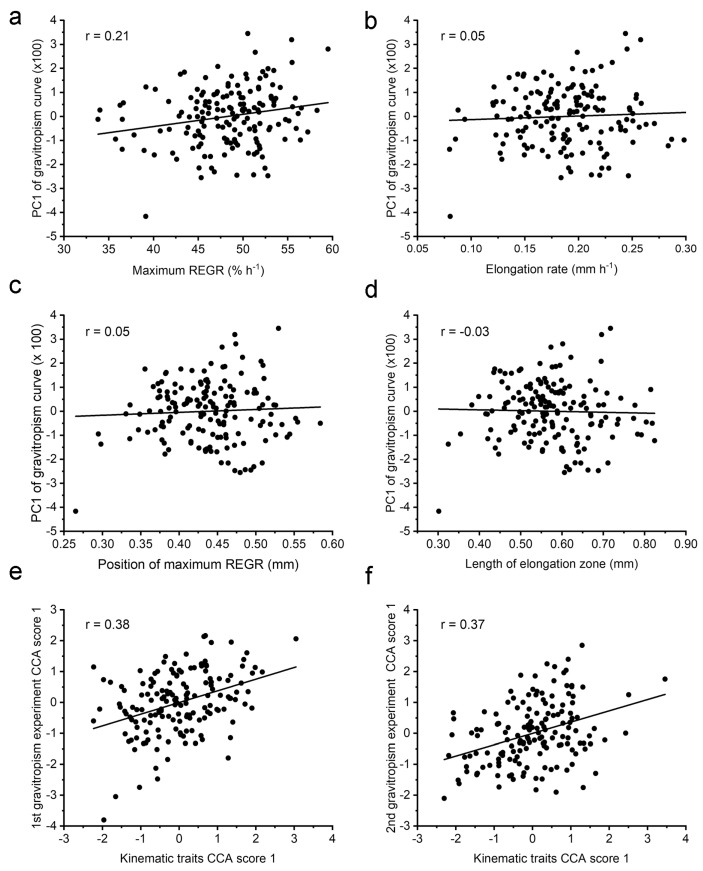
Correlations between each elongation zone trait and PC1 of gravitropism curves, and a canonical correlation analysis between all elongation zone traits and gravitropism data. (**a**) Gravitropism versus maximum REGR; (**b**) gravitropism versus elongation rate; (**c**) gravitropism versus position of maximum REGR; (**d**) gravitropism versus length of elongation zone; (**e**) canonical correlation analysis (CCA) score of the 1st gravitropism experiment versus the same for the set of four elongation zone traits; (**f**) same as (**e**) except the gravitropism data came from a second gravitropism trial.

The comparison in Figure 5 indicates that natural variation in a kinematic trait such as the length of the elongation zone or maximum REGR, assessed in a vertical seedling, do not share a genetic basis with natural variation in the bending growth during gravitropism, at least in this population. This conclusion predicts that an RIL’s kinematic traits will not correlate with its gravitropism response. To test this prediction, we reduced the gravitropism response curves to a single score (PC1) using principal components analysis. PC1 explains 68% of the variance in this population of curves, and the first three PC scores explain ~99% of the variance. PC1 was previously used to classify gravitropism response curves as belonging to one of three natural groups that shifted in their proportion of a population as a function of seedling age (2, 3, or 4 days old) or the size of the seed from which they emerged [21]. Because of its previously demonstrated utility as a descriptor, PC1 for each RIL was plotted against each of the kinematic traits for the same RIL to determine the degree of correlation (Figure 6a–d). Consistent with the QTL results in Figure 5, none of the kinematic traits correlated well with the PC1 of the gravitropism response curve. For example, Figure 6d shows that an RIL with an elongation zone that is 0.4 mm long and another with a 0.8 mm long zone may produce a similar difference in elongation rates between the upper and lower side in response to gravity. The *r* values for the four kinematic traits versus PC1 ranged from −0.03 to 0.21. The data used for these correlation analyses are presented in Supplemental File S1. Moore et al. [17] produced a second gravitropism dataset by repeating the measurements on seedlings derived from a separate seed source of the same RILs. Again, the growth zone traits did not correlate with PC1 of the tip angle curves obtained in this second gravitropism experiment. The *r* values ranged from −0.14 to −0.05. The data are presented in Supplemental File S1.

Another single measurement that can describe an important feature of a gravitropism response curve is the peak rate of change in tip angle, or maximum swing rate [21], which has also been called the tropic rate [23]. The maximum swing rate occurs when the elongation rate difference between the two sides of the root is the greatest. The average maximum swing rate for each RIL in the Moore et al. [17] gravitropism dataset was tested for correlation with each of the kinematic traits. The correlations were low, with *r* values ranging between 0.15 and 0.19. This result, the lack of overlapping QTL (Figure 5), and the low correlations in Figure 6a–d indicate that no single kinematic feature of the elongation zone, as determined in a vertically growing root, influences how differential elongation manifests during gravitropism.

The above correlation analyses treated each kinematic trait separately. They did not address the possibility that a combination of kinematic traits affects the ability of a root to produce a bend via differential elongation. Canonical correlation analysis, or CCA [24], was used to find a linear combination of kinematic traits that correlated with a linear combination of the first five PC scores of the tip angle response curves. The solution that CCA produces gives a score for the kinematics traits and a score for the gravitropism PCs. Figure 6e shows that scores correlate to the degree of *r* = 0.38. To determine if this value is significantly higher than one found by chance, 1000 permutations of the data were conducted. The 5% significance threshold derived from the permutation results was 0.35, so the correlation shown in Figure 6e is statistically significant. CCA was conducted to test for a relationship between combinations of the kinematic traits and the second set of gravitropism response curves that Moore et al. [17] obtained, which we call here Experiment 2. Figure 6f shows that the correlation between the two CCA scores was 0.37 and the significance threshold was again 0.35, as determined by the permutation method. Therefore, a combination of the kinematics traits can indicate to some extent how a root will bend in response to gravity (Figure 6e,f), although the same cannot be said about any single kinematic trait (Figure 6a–d). Table 2 shows the weights given to each of the kinematic traits in the CCA solution. The two separate gravitropism experiments produced a similar pattern. Maximum REGR received a high positive weight, while the rate of elongation received a large negative weight. The length of the elongation zone received a positive weight in both solutions. The position of maximum REGR was not consistent but also not as large. Therefore, both datasets produced a result that was statistically significant and generally similar in structure. The CCA method may produce a significant correlation but with unrealistic values. This can occur when a small feature of no biological significance in the data (noise) is greatly amplified by a large coefficient to achieve a spurious correlation. This was not the case here, as the factors in Table 2 are of the same order as the trait they are scaling. We guarded against the possibility of obtaining a meaningless correlation… by using only five PC scores to represent the gravitropism curves, and by testing the reliability of the result with permutation analyses. Thus, CCA produced a realistic and statistically significant result that indicates a gravitropic response is relatable to a weighted combination of the four kinematic traits, but not to any single trait (Figure 6a–d).

## 3. Discussion

The present work demonstrated that a recently described technical platform [14] can precisely measure the dynamic behavior of the Arabidopsis root elongation zone in a population suitable for genetic mapping. The throughput needed to make this application feasible was achieved by operating five microscopes in parallel, each recording growth for only one hour. Culturing seedlings on glass slides that fit into a custom sample holder with minimal handling also increased throughput by reducing the need for a lengthy growth-recovery period before initiation recovery, and it resulted in consistently clear images so failed trials were rare. Also, the Patch Track software is mostly automated so that post-acquisition analyses did not become a bottleneck. The quality of the data is a function of the clear texture in the images, the design of the Patch Track algorithm [14], and the degree to which the kinematic traits varied between RILs in the Cvi × L*er* population (Figure 1 and Figure 2). These factors were key to producing an unprecedented QTL model of the Arabidopsis root growth zone (Figure 3).

The apical and basal boundaries of the root elongation zone result from large changes in the rate of cell expansion occurring over very short distances. It is important to understand how such dramatic changes in expansion rates can be consistently established and controlled as new cells continuously traverse them. Presumably, some underlying axial gradient in physiological factors causes the cell walls to become abruptly more expandable at the apical boundary, and then less so at the basal boundary, Currently, auxin and cytokinin concentrations are thought to be two such factors, which together create a tip-high expression gradient in PLETHORA (PLT) transcription factors [25,26,27,28,29]. This then requires feedback regulation involving cytokinin signaling. The model maintains that cell division, elongation, and differentiation activities depend on how root cells “interpret local changes in the concentration of the PLTs” [30]. The kinematic results in Figure 1 and Figure 2 show that the position of the elongation zone can differ among the RILs by 0.3 mm, and that the length of the elongation zone ranges between 0.3 and 0.8 mm. These ranges correspond to several typical cell lengths. Experiments could test if the distribution of PLT expression varies between select RILs in a way that is consistent with the measured elongation zone parameters. A PLT-based model would be supported if, for example, PLT expression extended further basally in an RIL that displayed a long elongation zone, and less basally in an RIL that has a short elongation zone. However, if the PLT gradient was similar in two RILs that had distinctly different elongation zone lengths, then the role of PLT as a determinant of cell expansion capacity could be questioned. If such experiments support a PLT-based model, it would be reasonable to propose that the QTL intervals mapped here include genes that control PLT expression or function.

Auxin stimulates root elongation at very low concentrations but becomes inhibitory at higher concentrations [31]. Apparently, very low concentrations of auxin stimulate cell expansion by acidifying the apoplast, which promotes wall loosening, possibly by activating expansin proteins in the wall [32,33]. Therefore, future work could determine if RILs with large natural differences in kinematic traits display correspondingly different axial distributions of auxin signaling and apoplastic pH, which can be measured with fluorescent indicators. The results of Henry et al. [14] indicate such experiments could be informative because mutations in three transporters known to influence auxin distribution in roots (PIN2, ABCB4, and ABC19) affected the elongation zone. The results were interpreted as evidence that PIN2 shapes the leading edge of the elongation zone by delivering growth-suppressing (supra-optimal) amounts of auxin to the apical end of the elongation zone, which would explain why a *pin2* mutation shortens the meristem [34,35], and that ABCB transporters reduce auxin concentrations more basally to the extent that it becomes expansion-limiting and contributes to the REGR decline in the shootward side of the elongation zone. The QTL discovered here may control the auxin gradient and/or the shape of the biphasic auxin sensitivity (dose–response) curve, which PLTs may control.

The size, shape, and position of the elongation zone is probably not solely the result of an axial auxin gradient, a biphasic (bell-shaped) auxin dose–response curve, and an axial PLT gradient. At the very least, a complete description of the mechanism would have to include a role for cytokinin, as this hormone plays a role in the PLT mode of action [28,29,36]. Lee et al. [37] measured cytokinin levels in roots in the same Cvi × L*er* RIL population used here. Intriguingly, a QTL that explained variation in the levels of zeatin (a type of cytokinin) and two zeatin glucosides appears to directly match QTL 2@47.6 for the length of the elongation zone (Figure 3; Table 1). Natural variation in a yet-unidentified gene at this locus appears to affect the levels of cytokinin and cytokinin metabolites, and this genetic variation may also cause a variation in the length of the elongation zone in the Cvi × L*er* RIL population.

Cytokinin has been shown to promote cell differentiation and decrease the number of meristematic cells to help establish the transition from the meristematic to the elongation zone [30,36,38]. Also, cytokinin can promote cell expansion by promoting ARR1-dependent activation of an expansin and two proton pumps, which play well-understood roles in controlling cell expansion [33,39]. Thus, a gene that controls cytokinin levels in roots might also cause a change in elongation zone length. Future work could use fluorescent reporters of cytokinin signaling [40] in select RILs to determine if any change in patterns could explain the positions of the elongation zone boundaries quantified by kinematic analysis.

The kinematic traits of a root elongation zone are dynamically controlled. When measured continuously for 3 h with the Stripflow kinematic method, Baskin et al. [41] discovered that the elongation zone of the Arabidopsis primary root moves back and forth (saltates) several microns every 5 min, which they interpreted to be evidence that an endogenous feedback mechanism continuously balances the position of the zone. The control mechanism may involve a plasma membrane receptor kinase because kinematic analysis revealed erratic REGR patterns in the elongation zone of *feronia* mutants [11]. Another control mechanism in Arabidopsis and maize maintains a constant elongation zone length, while temperature increases raise the maximum REGR [42,43]. Although temperature does not alter the length of the elongation zone in Arabidopsis, water potential does in maize seedlings. Decreasing water potential shortens the elongation zone of maize primary roots by inhibiting REGR only in the basal half of the primary root’s elongation zone [44,45].

Gravity is another environmental signal that must adjust kinematic parameters when it induces bending growth, but which kinematic traits change in cells that are separated by only the width of the root is not known. Although the kinematic basis of root gravitropism may not have been studied in detail, the bending itself is proof of the very fine-scale adjustment of kinematic parameters within the elongation zone. We hypothesized that one or more of the kinematic parameters determined by an RIL’s genotype would affect its bending response. For example, we thought it was likely that RILs with high maximum REGR would respond similarly to gravity, and differently from RILs with low maximum REGR. If true, then maximum REGR should correlate with a quantitative measure of gravitropism. Also, in this hypothetical scenario, shared QTL for maximum REGR and gravitropism would be expected. The results in Figure 5 and Figure 6a–d do not support this hypothesis. Instead, the kinematic adjustments that cause an Arabidopsis root to bend in response to gravity appear not to depend on the same genetic elements that affect any single elongation zone trait determined in a vertically growing root. Major gravitropism QTL are not also QTL for any of the elongation zone kinematic traits (Figure 5), and RILs with very dissimilar values for any of the kinematic traits could produce similar bending responses in response to gravity (Figure 6a–d). These results may be evidence that the gravitropism QTL [14] affect the gravity signaling process rather than the kinematic adjustments that create bending. None of the new gravitropism genes that Yoshihara et al. [46] found at the gravitropism QTL that Moore et al. [14] mapped are known cell expansion regulators, consistent with this reasoning. Also, QTL for root skewing, a type of bending growth on backward-inclined surfaces, do not co-align with QTL for the growth zone traits mapped here [20].

The present results, which may apply only to this Cvi × L*er* population, indicate that natural variation in a single kinematic parameter such as elongation zone length is not responsible for natural variation in gravitropic bending. However, a deeper analysis indicated that some relationship exists between the elongation zone kinematics and gravitropism. The results in Figure 6e,f and Table 2 indicate that an equation can convert a weighted combination of the four kinematic traits that an RIL possesses into a tip angle curve that would develop after a 90 degree gravity stimulus. The results in Table 2 show that the two solutions found by the CCA method were similar in form. Maximum REGR and elongation rate were weighted most heavily and consistently. The emphasis on elongation rate may be related to the positive relationship Durham Brooks et al. [21] found between elongation rate during gravitropism and PC1 of the tip angle curve for Col-0 wild-type seedlings.

The QTL mapped here could lead to the identification of components that determine how material so profoundly changes at the meristem/elongation zone boundary, how its local strain rate (REGR) can so quickly reach rates above 50% h^−1^, and what causes it to decrease again almost as abruptly at the differentiation zone. Also, the finding that two RILs can differ more than two-fold in kinematic traits like elongation zone length could be leveraged to test current molecular gradient-based models of root zonation using natural variation rather than knockout mutants as the genetic variable. Plant growth kinematics has heretofore not been a high-throughput field, but this work demonstrates that dynamic phenotypes requiring measurements on submicron spatial scales and temporal scales of minutes are feasible even when large numbers of trials are necessary. Thus, quantitative genetics can now be used in combination with molecular genetics and cell biology to investigate the root growth engine.

## 4. Materials and Methods

### 4.1. Germplasm and Image Analysis

Recombinant inbred lines derived from a Cvi × L*er* cross [18] were used to map QTL. Of the 162 lines in this RIL population, RILs 5, 89, and 115 were not included in the analysis due to lack of germination. All plant growth conditions, imaging, and kinematic analyses in this work were as previously described [14]. For each RIL, 7–10 independent trials were performed.

### 4.2. Quantitative Trait Loci Analysis

The quantitative trait loci map was constructed using the R/qtl2 package [47]. The phenotypes were the kinematic traits obtained from the image analysis pipeline. The marker data and genetic maps were provided for the Cvi × L*er* RIL population using 234 amplified fragment-length polymorphism-based markers [18]. Missing genotype data were accounted for using the hidden Markov model, pseudomarkers were inserted every 1 cM, and a genotyping error rate of 0.001 was assumed [48]. The genome was scanned using a one-dimensional, single QTL model using Haley–Knott regression [49]. To determine the 5% significance threshold, 25,000 permutations were performed [19], and then a 1.5 LOD support interval was used to define the confidence intervals at each locus.

### 4.3. Correlation Data Analysis

Principal component analysis (PCA) was performed with the statistics package available in R [50]. Canonical correlation analysis (CCA) was performed with the CCA package available in R [51]. All the traits were z-score-normalized before performing CCA.

## 5. Conclusions

A previously described machine vision platform and associated computational pipeline for the kinematic analysis of Arabidopsis roots can serve as a phenotyping tool in large-scale genetic studies. The tool was used to identify several regions in the Arabidopsis genome that control kinematic traits such as the length of the elongation zone, the position of it, and its maximum rate of local expansion (REGR). Some of these genetic loci (QTL) were trait-specific while some others controlled more than one trait. The kinematic traits and previously determined gravitropism phenotypes were mapped, almost without exception, to different regions of the genome. This result and other correlation analyses indicate that the generation of differential growth during gravitropism is not predetermined by any single characteristic of the elongation zone.

## Figures and Tables

**Table 1:** Significant QTL positions by growth zone trait. Rows indicate the chromosomal location and trait associated with the QTL.

Trait	Chromosome	Position (cm)
Rate of elongation	1	21.5
Position of maximum REGR	1	21.5
Length of elongation zone	1	21.5
Length of elongation zone	1	76.2
Rate of elongation	1	77.9
Length of elongation zone	2	47.6
Length of elongation zone	3	4.8
Maximum REGR	3	39.7
Maximum REGR	5	76.7
Rate of elongation	5	76.7

**Table 2:** Factor values (unitless) from a canonical correlation analysis showing the weight placed on each elongation zone trait to achieve a significant correlation with principal components of gravitropism tip–angle curves from the Cvi × L*er* population of RILs measured in two independent experiments.

Experiment	Maximum REGR	Rate of Elongation	Position of Maximum REGR	Length of Elongation Zone
1	2.5235	−3.9280	0.5345	1.5952
2	3.1872	−5.3127	−1.1253	4.4978

## Data Availability

All phenotype data are presented in Supplemental File S1.

## Supplementary Materials

The following supporting information can be downloaded at https://www.mdpi.com/article/10.3390/plants13091189/s1, Figure S1: Parameter sweeps show how *v_f_*, *x*_0_, *k*, and *n* in the flexible logistics equation affect the size, shape, and position of the elongation zone; File S1: The velocity profiles for each RIL used to perform the QTL mapping; File S2: The velocity profiles for each RIL used to perform the QTL mapping; Video S1: 30 min of an experimental recording played forwards and backwards to highlight where cell expansion occurs.
