# Supplementary figures and images for: QTL for the Kinematic Traits That Define the Arabidopsis Root Elongation Zone and Their Relationship to Gravitropism
Source: Plants (Basel). 2024 Apr 25;13(9):1189. doi: 10.3390/plants13091189 (PMC11085590; doi:10.3390/plants13091189)

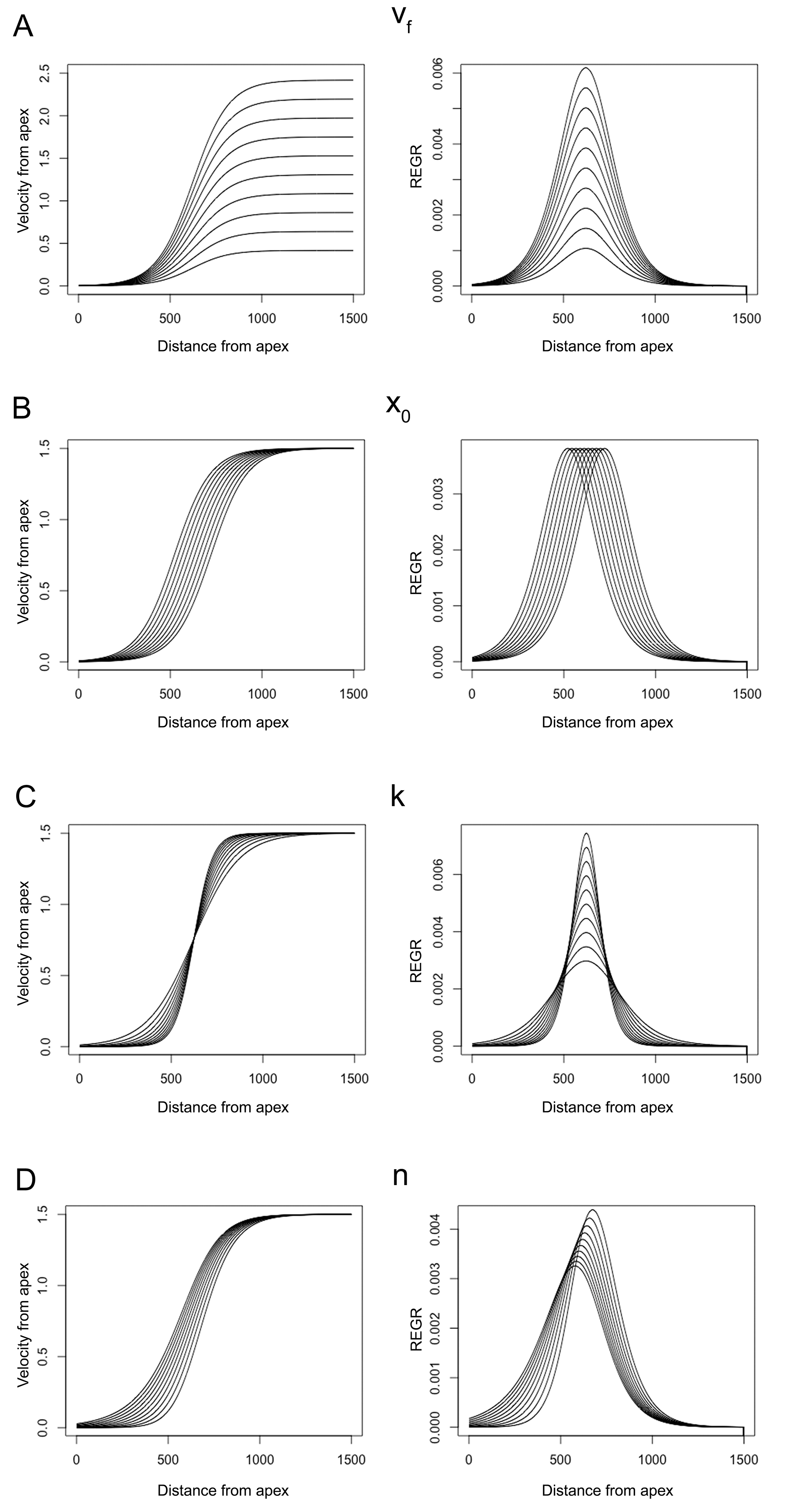

Supplement: Supplementary file 1 [file plants-13-01189-s001.zip › FigureS1.tif]
